# Further decoding the mystery of American pain: The importance of work

**DOI:** 10.1371/journal.pone.0261891

**Published:** 2022-01-13

**Authors:** David G. Blanchflower, Alex Bryson

**Affiliations:** 1 Department of Economics, Dartmouth College, Hanover, New Hampshire, United States of America; 2 Social Research Institute, University of London, London, England; TED University, TURKEY

## Abstract

A recent paper showed that, whereas we expect pain to rise with age due to accumulated injury, physical wear and tear, and disease, the elderly in America report less pain than those in midlife. Further exploration revealed this pattern was confined to the less educated. The authors called this the ‘mystery of American pain’ since pain appears to rise with age in other countries irrespective of education. Revisiting this issue with the same cross-sectional data we show that what matters in explaining pain through to age 65 is whether one is working or not. The incidence of pain across the life-course is nearly identical for workers in America and elsewhere, but it is greater for non-working Americans than it is for non-workers elsewhere. As in other countries, pain is hump-shaped in age among those Americans out of work but rises a little over the life-course for those in work. Furthermore, these patterns are apparent *within* educational groups. We show that, if one ascribes age-specific employment rates from other OECD countries to Americans, the age profile of pain in the United States is more similar to that found elsewhere in the OECD. This is because employment rates are lower in the United States than elsewhere between ages 30 and 60: the simulation reduces the pain contribution of these non-workers to overall pain in America, so it looks somewhat similar to pain elsewhere. We conclude that what matters in explaining pain over the life-course is whether one is working or not and once that is accounted for, the patterns are consistent across the United States and the rest of the OECD.

## Significance statement

Recent research [1] points to a ‘mystery’ in American pain since, contrary to expectations, it is hump-shaped in age for the less educated, a pattern not observed elsewhere. We show differences in the pain experienced by non-workers, together with the age-employment profiles between the United States and elsewhere account for the ‘mystery’ in American pain. As in other countries, it is hump-shaped in age among those out of work but rises a little over the life-course for those in work. These patterns are apparent *within* educational groups. What drives the difference in the age profile of pain is higher pain incidence among non-workers in America compared to elsewhere and differences in employment rates between the United States and the rest of the OECD. Employment rates in the United States have been falling faster than anywhere else in the last two decades (S1 Table). Since employment rates in the United States are lower than those in the rest of the OECD across most of the life-course, and employment rates are falling, fixing the pain problem in America requires fixing the labor market for the left-behinds.

## Introduction

There is a high level of pain among Americans leading to increased opioid use [2] and “deaths of despair” from suicides, drugs and alcoholic liver disease [3–5]. In one study in 2012 126 million, or 56% of American adults, reported experiencing some sort of pain. Of these 20% had daily pain [6]. These pain problems, Case et al. [1] argue, are “*concentrated among Americans with less than a bachelor’s degree*” (p. 24785). They find that, among this less educated group, each successive birth cohort has a higher prevalence of pain at each point in the lifecycle, whereas this is not the case for degree-holders. Higher pain among later-born cohorts of those without a bachelor’s degree explains why, in cross-sectional data, elderly Americans are in less pain than those in midlife. Among those with at least a bachelor’s degree, on the other hand, pain rises with age, as one might expect due to the accumulation of injury, physical wear and tear and disease.

In an earlier time-use survey Krueger and Stone [7] found Americans with lower income and less education spent a higher proportion of time in pain and reported higher average pain than did those with higher income or more education. However, in Krueger’s [2] later work the focus was not on the link between pain and education, but that between pain and labor market participation. Whilst the causal relationship between pain and labor market participation is contested in the literature [8] Krueger [2] finds pain incidence is twice as high among men not in the labor force (NILF) compared with men in the labor force which includes both the employed and the unemployed. There is a similar, though slightly smaller differential, among women. Nearly half of NILF prime-age men are found to take daily pain medication. Krueger suggests pain, and the prescription of opioids, has played a key role in the decline of labor force participation in America in recent decades “*causing the problem of depressed labor force participation and the opioid crisis to become intertwined*” (p. 1).

We re-examine the experience of pain through age sixty-five using the Gallup Daily Tracker Survey (2009–2017)–henceforth GUSDT–in the United States with 2,279,477 unweighted observations and 1,589,152 observations for those ages 18–65. We also make use of a further twenty OECD countries taken from the Gallup World Poll (2009–2020)–henceforth GWP–with 311,669 observations, the data files and list of countries used by Case et al. [1]. The OECD 20 country sample comprises sixteen major European countries—Austria; Belgium; Denmark; Finland; France; Germany; Iceland; Ireland; Italy; Luxembourg; Netherlands; Norway; Spain; Sweden; Switzerland and the UK—plus Australia, Canada, Japan and New Zealand. There are 240,287 observations on pain available for analysis for those age 15–65. We use the cutoff of 15 for the OECD countries because of lower school leaving ages in some OECD countries.

The question asked in both surveys was:

*“Did you experience the following feelings during a lot of the day yesterday? How about physical pain? Yes/no”*

Earlier papers using these Gallup data have analyzed pain [9–13]. We confine our analyses to those aged between eighteen and sixty-five years in the USA and fifteen through sixty-five in the OECD, partly because there is substantial mortality selection in well-being after age 65 [14] and in part because paid work–which is our main focus–is far less common in the over 65s. The extent to which this is true, of course, varies by country.

We focus on cross-sectional variance rather than variance across birth cohorts. We have insufficient data over time to distinguish between age and cohort effects. We show that what matters in explaining pain over the life-course is whether one is working or not. The hump-shape in pain with age is apparent among non-workers in America and elsewhere. It is absent among workers in America and elsewhere. Furthermore, these patterns are apparent *within* educational groups.

If one ascribes age-specific employment rates from other OECD countries to Americans, the age profile of pain in the United States is more similar to that found elsewhere in the OECD. We conclude that what matters in explaining pain over the life-course is whether one is working or not and that this is apparent across countries. In this sense our results reflect Krueger’s [2] study which emphasized the importance of work. The ‘mystery of American pain’ is partly accounted for once age-employment rates across countries are adjusted. Nevertheless, Case et al. [1] are right to emphasize the problem of American pain, particularly among non-workers: it affects millions of Americans and deserves the attention of both the academic and policy communities [15].

## Results

Pain rates in the United States are high [10]. Our own analysis of pain data for the United States and 20 other OECD countries in the GWP confirms pain rates in the US are high and comparable to France, Spain and Canada but above Germany, and the UK (Table 1 Panel a)). (Pain rates in the GUSDT are a little lower averaging 24% versus 28% for the GWP which has a much smaller sample size). We report data for the USA which is also available in the Gallup World Poll and rank by pain level. The exact question asked in both surveys is "*Did you experience physical pain yesterday*–yes or no?"

**Table 1:** Pain rates in the gallup world poll, 2009–2020 and gallup US tracker poll, 2009–2017.

a) By country, 2009–2020 (n = 311,669)		
Iceland	32.1	
Belgium	29.6	
France	29.3	
Spain	29.3	
USA (n = 15,243)	28.3	
Canada	27.9	
Luxembourg	25.8	
Italy	25.2	
Denmark	24.7	
Australia	23.9	
Total	23.8	
Finland	23.7	
Switzerland	23.6	
New Zealand	23.0	
Germany	22.5	
Norway	22.5	
Sweden	21.5	
Austria	21.3	
United Kingdom	21.1	
Netherlands	20.9	
Japan	19.7	
Ireland	19.2	
b) By year		
	OECD (n = 311,669)	USA (n = 2,279,477)
2005	22.0	
2006	22.1	
2007	22.7	
2008	22.4	
2009	24.2	23.9
2010	22.2	23.8
2011	22.3	24.1
2012	23.4	23.9
2013	26.0	24.6
2014	25.2	24.7
2015	23.2	24.8
2016	25.2	24.8
2017	24.7	27.1
2018	24.1	
2019	25.9	
2020	25.5	

Iceland has the highest pain rate with the United States fifth. We also report time series changes in pain rates over time. In the first column of Table 1 Panel b) we also report OECD estimates for 20005–2008 that we exclude from our subsequent analysis due to the lack of a comparable work variable. It is apparent that there was a small rise in pain after 2005–2008. In subsequent analyses we focus on pain rate after 2008 which is the period during which we have a comparable labor market variable. In contrast to the OECD the US shows a rise in pain with an especially high rate of 27.1 (n = 160,226) in 2017. We should note that this average rate from GUSDT (23.8%) is somewhat lower than in part a) of Table 1 with a smaller sample (28.3%), and for more years, through 2020 rather than 2017.

**Fig 1:**
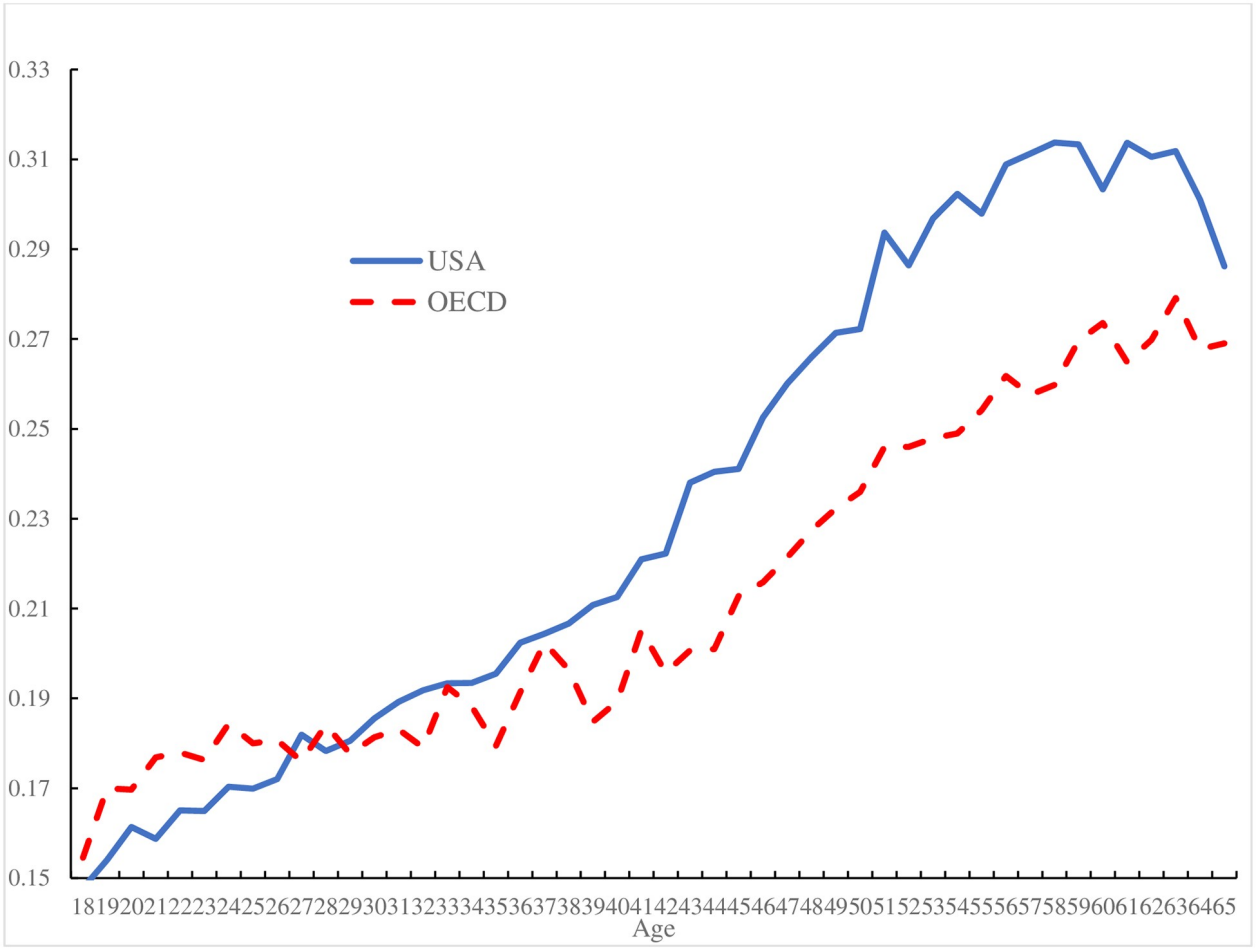
Pain by age in the United States (2009–2017) and the OECD (2009–2020).

Fig 1 plots the prevalence of self-reported pain against age in America and the OECD which is the starting point for Case et al’s [1] paper. They confine their analyses to white and black non-Hispanics aged 25–79 years between 2008 and 2017. We see no reason to exclude other minorities so throughout this paper our analyses are based on Americans of all races and we include those under 25, who are especially happy [16], and truncate our analyses at age 65 for two reasons. First, our focus is on the role of paid work, an activity which falls markedly after age 65 with retirement. Second, we reduce the possibility that our results are affected by truncation in the sample arising from “deaths of despair” which, as Case and Deaton [4] explain, are most prevalent among the less educated suffering pain. The results replicate Case et al.’s [1] in showing pain rising with age in the rest of the OECD, whereas it is hump-shaped in the United States.

**Fig 2:**
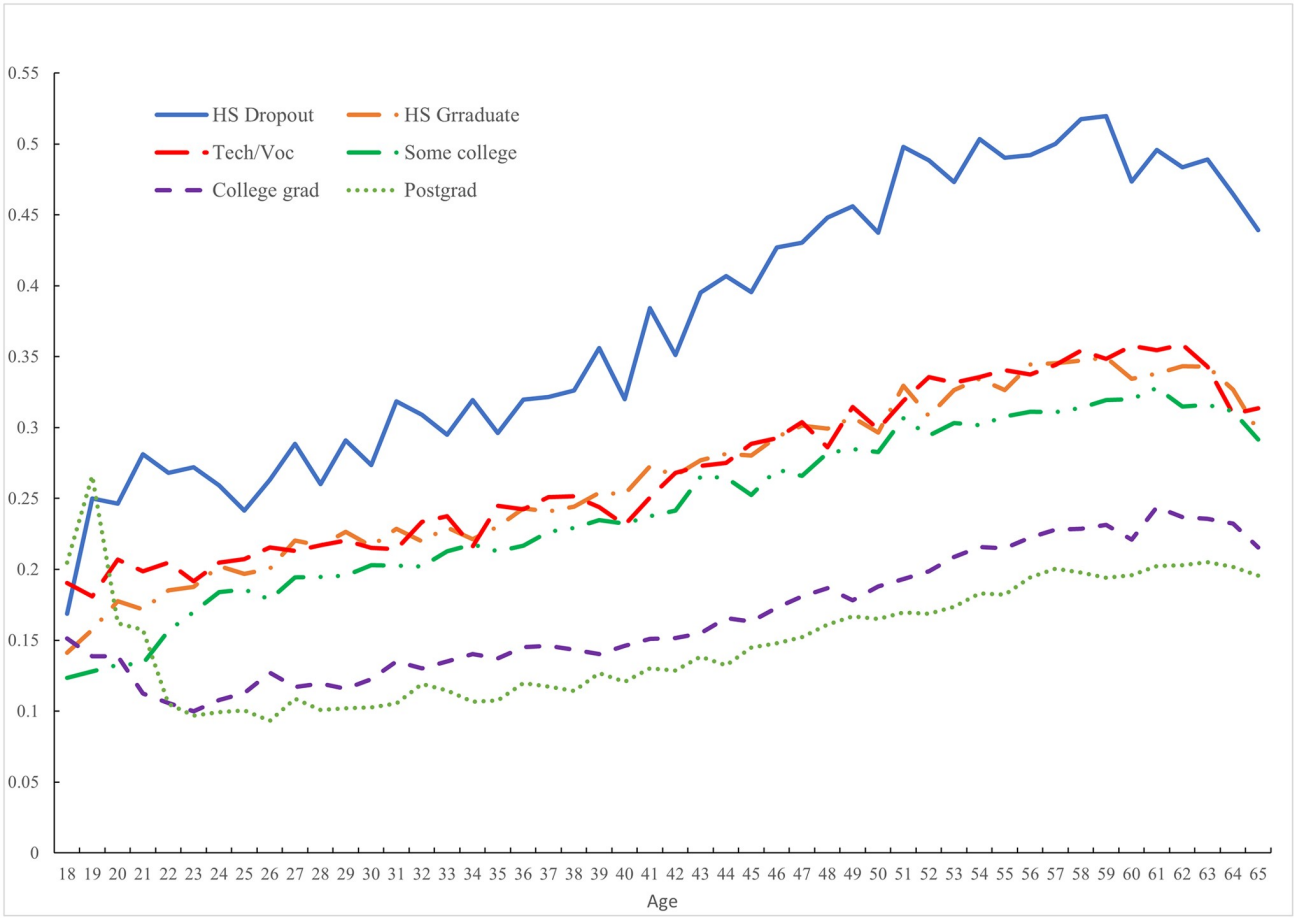
Pain in the USA by education group, 2009–2017.

Fig 2 plots the same data for the United States but presents the pain data by education. There are hump-shapes in pain with age among those with education below degree level, whereas pain rises gradually with age among those holding bachelors’ and postgraduate degrees. Pain peaks in one’s late-50s among high school dropouts, over half of whom report pain. In contrast, no more than one-quarter of degree holders ever report pain, with the proportion rising gradually with age.

**Fig 3:**
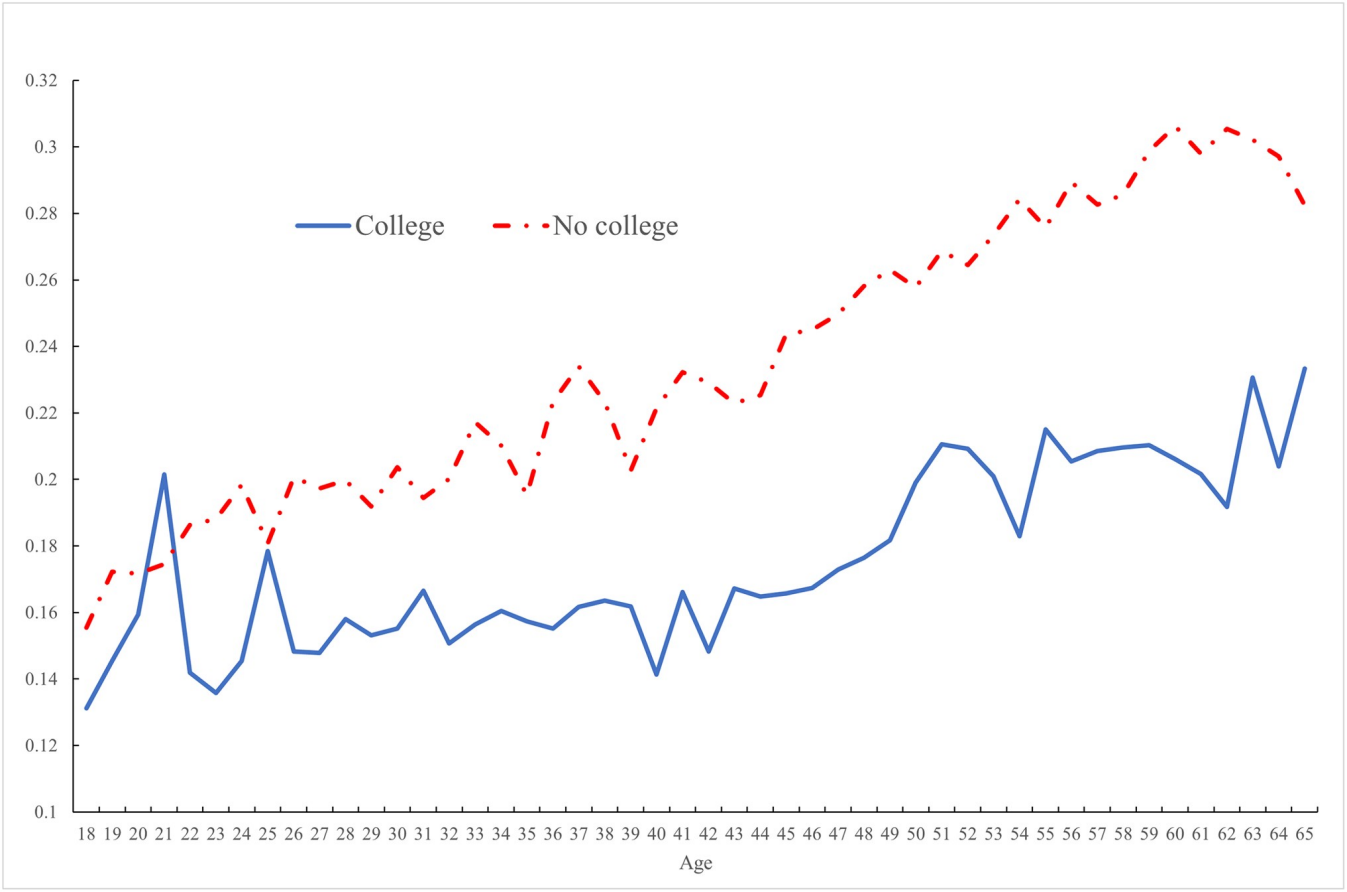
Pain in OECD 20 countries by education group, 2009–2020.

In contrast Fig 3 plots average (mean) pain by age for 20 other advanced countries in the OECD for those with and without college education. These countries are: Australia, Austria, Belgium, Canada, Denmark, Finland, France, Germany, Iceland, Ireland, Italy, Japan, Luxembourg, Netherlands, New Zealand, Norway, Spain, Sweden, Switzerland, and the U.K Pain incidence rises with age, whether one is college educated or not, but it is lower for college graduates.

These figures replicate those that are the basis for Case et al.’s [1] claim that the United States is different from other advanced countries in the OECD. Below we show that the age-profile of pain among the working-age population in the United States and other OECD countries is similar once one accounts for differences in employment rates by age across the United States and the OECD. Following Krueger [2] we examine differences in pain depending on whether the respondent was working or not working.

**Fig 4:**
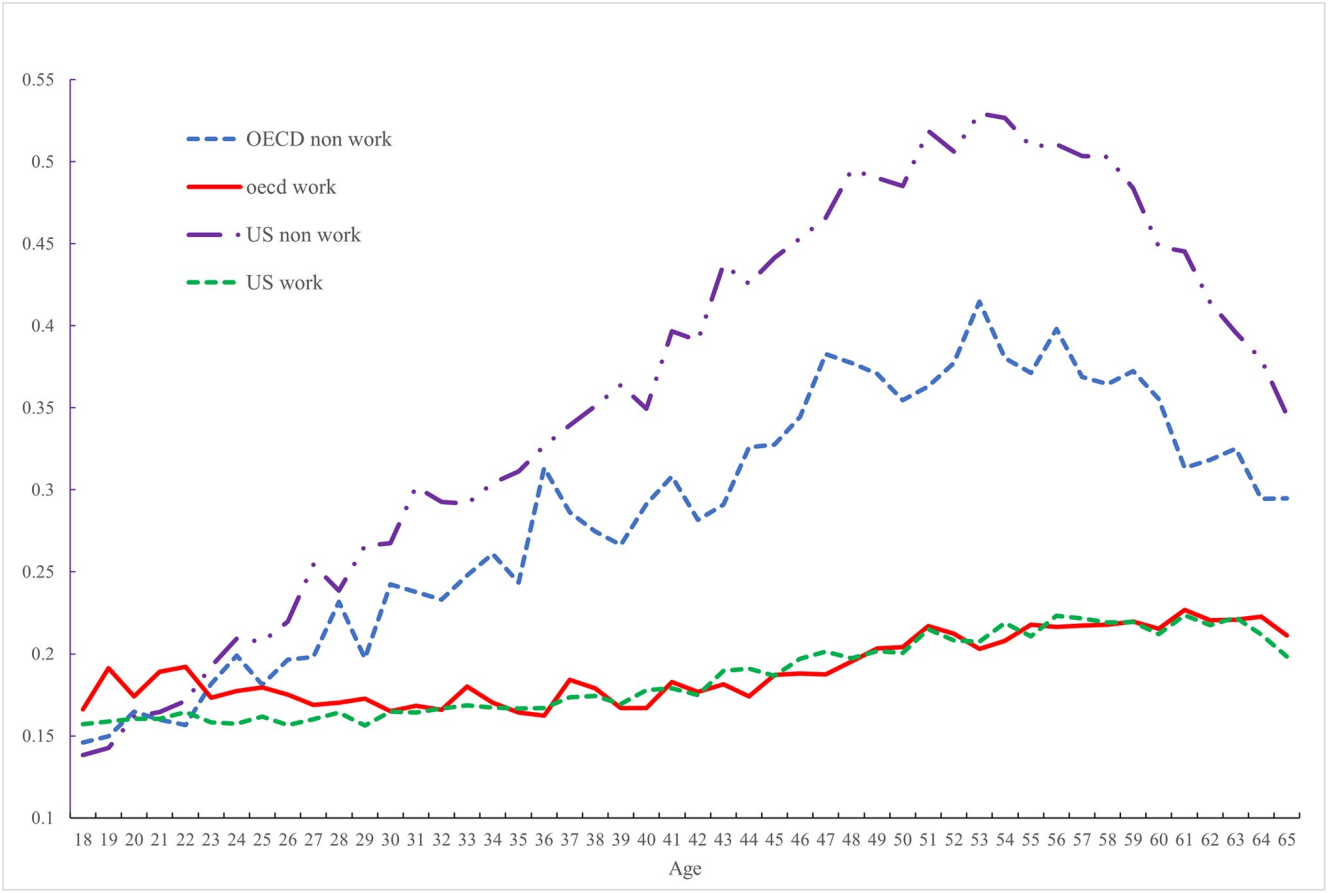
Pain in the OECD (2009–2020) and USA (2009–2017) for workers and non-workers.

To show the centrality of paid work in understanding the age-profile of pain Fig 4 presents the data underlying Figs 2 and 3, but this time plots age-profiles by working status when the work data are collected in a consistent fashion. Pain incidence is almost indistinguishable between workers in America and elsewhere once people enter their 30s and rises gently as they age. In stark contrast pain is hump-shaped in age in both the United States and the rest of the OECD, peaking in one’s early 50s and declining subsequently. But throughout the life-course, the prevalence of pain among non-workers is far greater among Americans than it is elsewhere in the OECD. By their early 50s over half of all non-working Americans report pain compared with two-in-five of those elsewhere in in the OECD.

**Fig 5:**
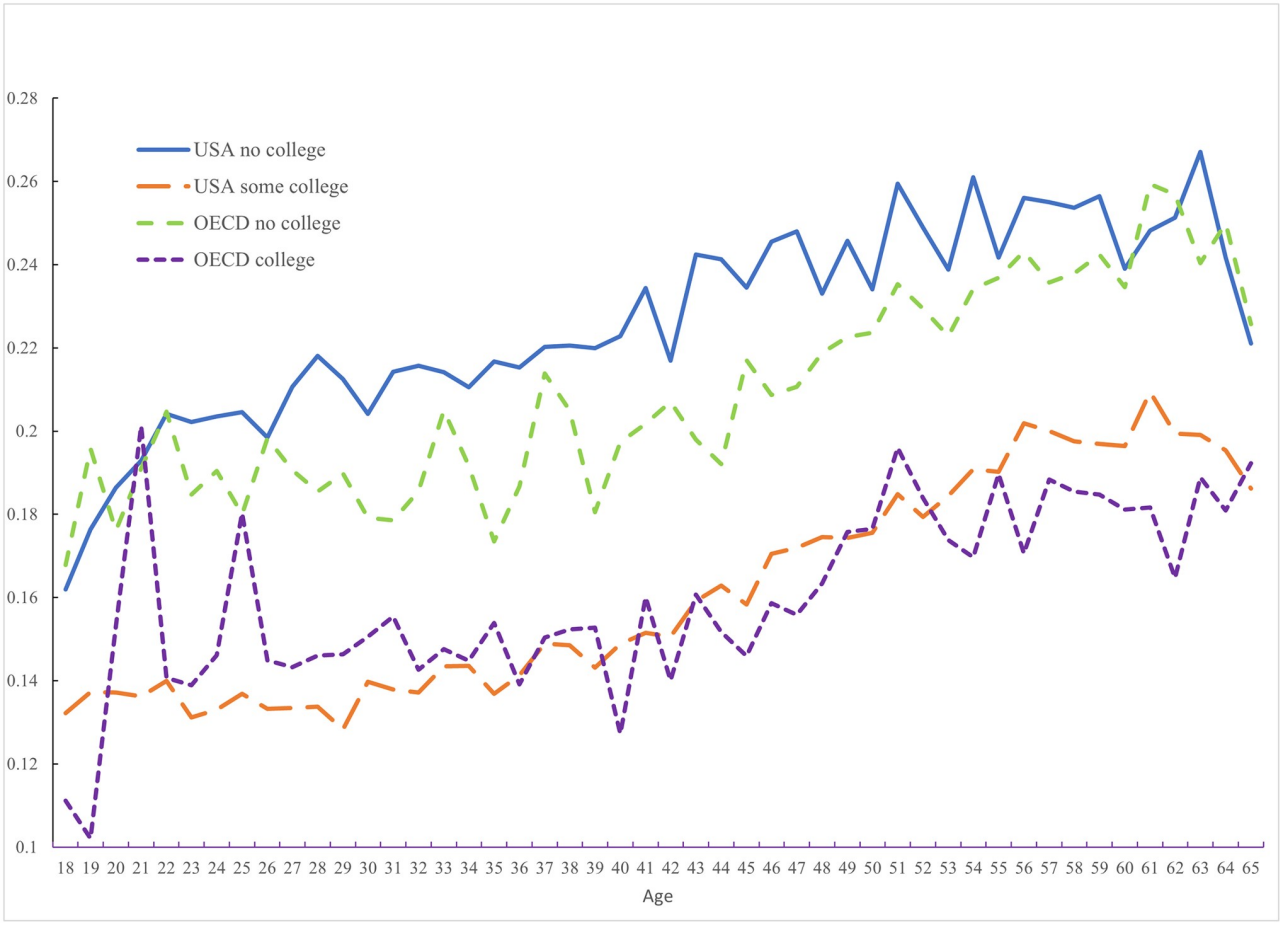
Pain among workers by education in the USA (2009–2017) and OECD (2009–2020).

Fig 5 shows that pain among workers rises with age in both the United States and in other OECD countries and that this is the case whether one is college educated or not. College educated workers experience far less pain than non-college educated workers both in the United States and elsewhere, but the pain-age profile for workers is similar when conditioning on education, both in the United States and elsewhere.

**Fig 6:**
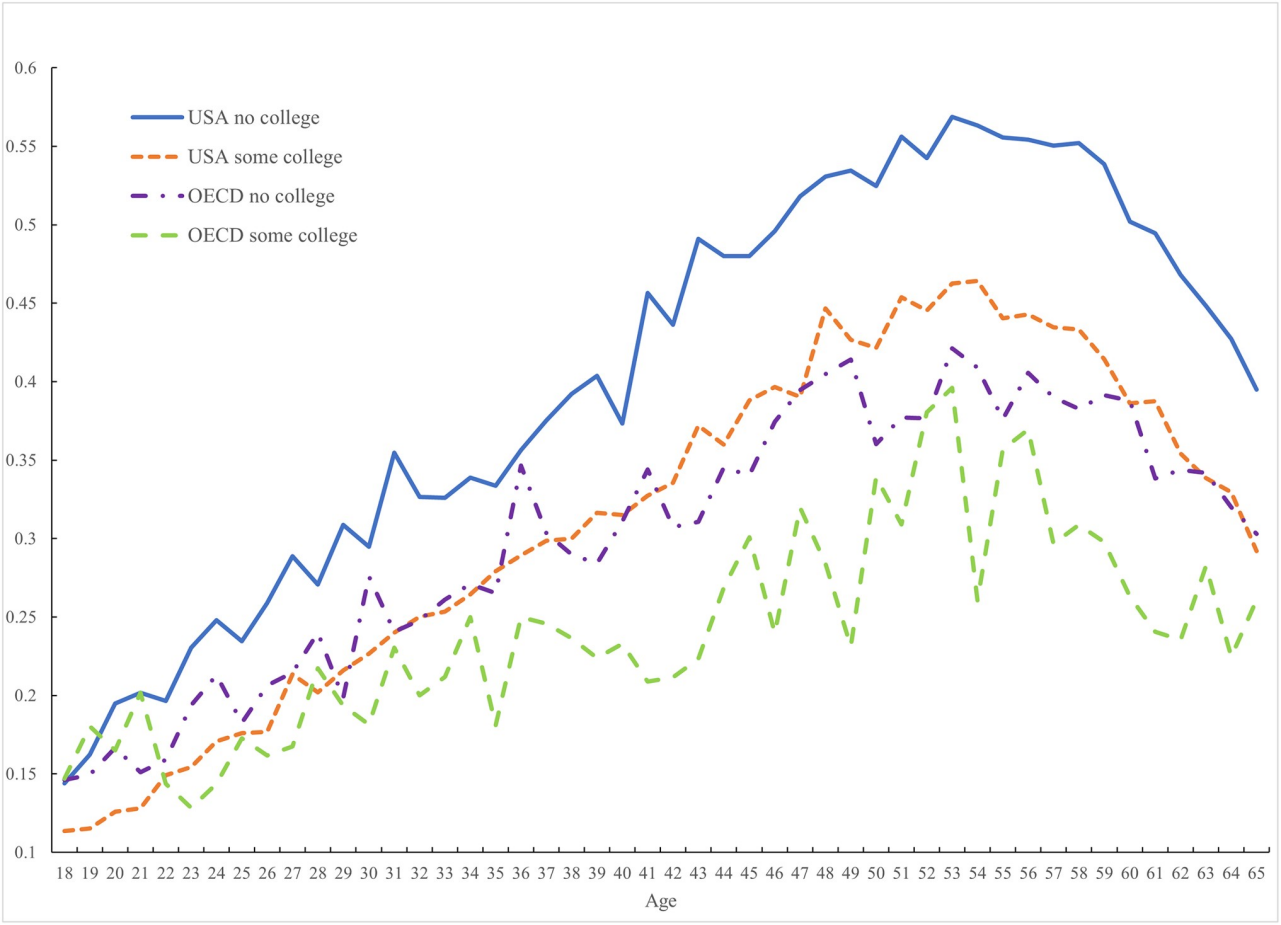
Pain among non-workers by education in the USA (2009–2017) and OECD (2009–2020).

The hump shape in pain among non-workers is evident in both the United States and the rest of the OECD in Fig 6. It is apparent whether one is college educated or not. However, the hump shape is much more pronounced in the United States and pain incidence among non-workers is considerably greater than elsewhere both for college educated and non-college educated non-workers.

We have shown that the age profile of pain, both in the United States and elsewhere in the OECD, is strongly correlated with individuals’ employment status and that, once one divides people into workers and non-workers, the age-profile in pain is similar for the United States and the rest of the OECD. And yet the age-profile of pain in the aggregate differs as between the United States and other OECD countries. As Case et al. [1] noted it is hump shaped in the United States but rising in age in the rest of the OECD. This is also the case among those aged 65 and under (Fig 1).

The reason for this difference is related to differences in the pain experienced by non-workers in the United States and elsewhere together with differences in age-employment profiles in the United States and the rest of the OECD. S1 Fig plots by single year of age the percent of the age group that is working. Employment rates are lower in the OECD than they are in the United States until people reach their late-20s. From that point until people reach 60 employment rates are higher elsewhere in the OECD than they are in the United States, after which Americans are more likely to be in employment than their counterparts elsewhere.

**Fig 7:**
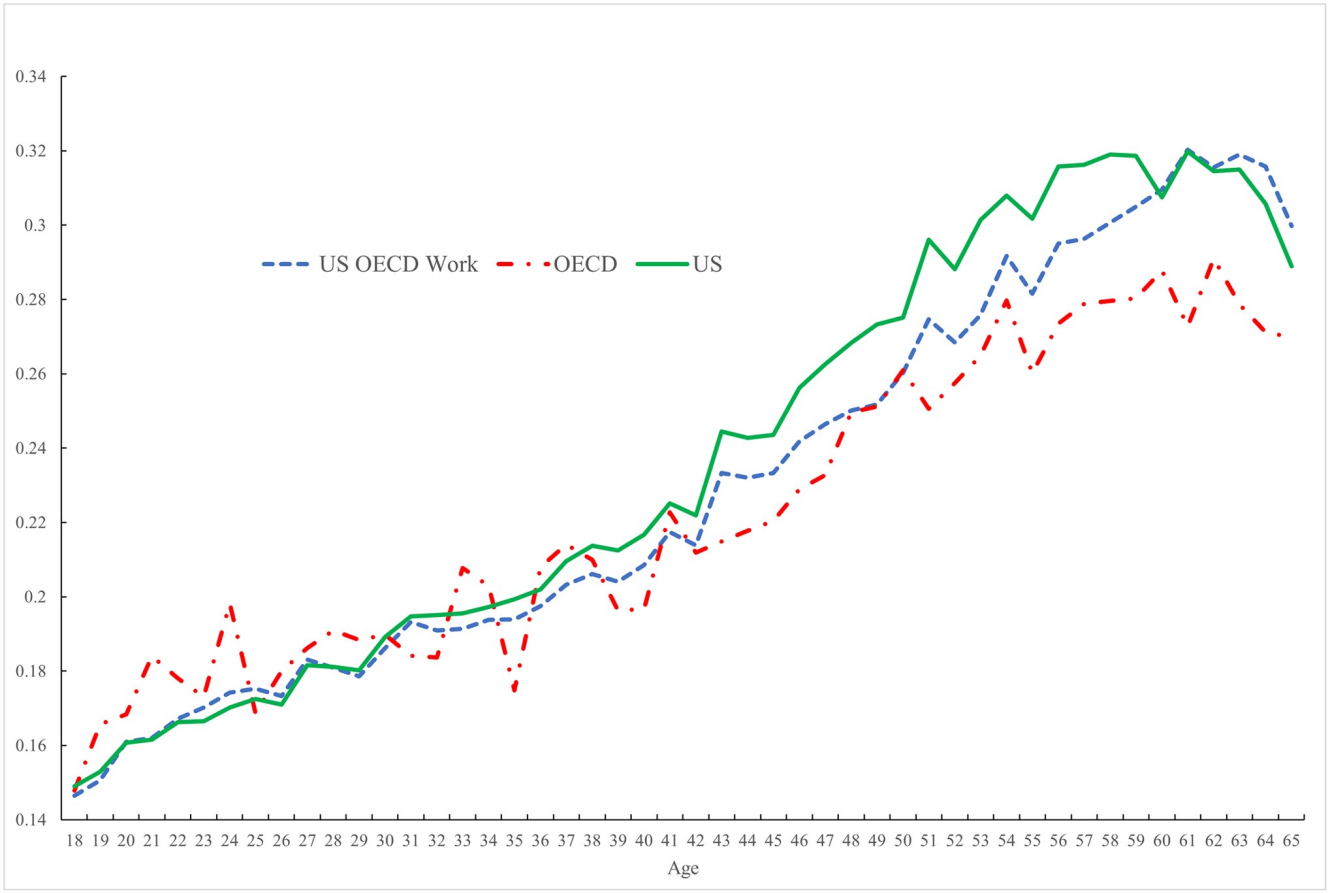
Simulation of age-pain profile in the United States having adjusted the age-employment (2009–2020).

The implications of these differences in employment rates across the lifecycle for the incidence of pain in the United States and the rest of the OECD are depicted graphically in Fig 7. The figure reports the aggregate age-pain profiles for Americans (the green line) and the rest of the OECD (the red dotted line), confirming the hump-shaped nature of pain in the United States. In contrast, as Case et al. [1] note, pain tends to rise with age in the rest of the OECD, although it does flatten out when they reach their late-50s. We have added a third line to this figure: this dotted blue line redraws the age-pain profile in America, but this time rather than using American employment rates, it recalculates US pain using OECD rather than US employment rates. This adjustment lowers the pain incidence in the United States for those in their 30s through to age 60 such that the age-pain profile looks more similar to that in the rest of the OECD. The adjustment reduces the contribution of non-workers to the pain incidence in the United States and, since that is particularly high, it reduces the pain experienced by Americans in the simulation until they reach age 60. At this point employment rates are higher in America than elsewhere, so the adjustment does nothing to change pain incidence in the American population.

From their mid-50s onwards the pain experienced by Americans exceeds that experienced by those in the rest of the OECD, whether we adjust employment rates between the OECD and the United States or not. Table 2 sheds light on why this is the case by showing what happens to the incidence of pain by single year of age from 56–65 in both the United States and the rest of the OECD.

**Table 2:** Pain simulated for non-workers by age, USA (2009–2017) and OECD20 (2005–2020).

a) USA				
Age	Non workers	Workers	% work	All % in pain
	% in pain	% in pain		
56	51	22	68	31
57	50	22	67	31
58	50	22	65	32
59	48	22	63	31
60	45	21	60	31
61	45	22	57	32
62	41	22	51	31
63	40	22	48	31
64	38	21	45	30
65	35	20	39	29
b) OECD20				
Age	Non workers	Workers	% work	All % in pain
	% in pain	% in pain		
56	39	22	72	26
57	39	23	71	26
58	37	22	69	26
59	40	22	65	27
60	37	23	57	27
61	32	24	53	26
62	34	24	47	27
63	34	23	42	27
64	31	24	36	26
65	31	23	29	27

The first column shows the age of individuals. Columns 2 and 3 present the percentage of non-workers and workers respectively who experience pain. Column 4 is the percentage working. The fifth column reports the percentage of the whole population experiencing pain.

Patterns in these data are very similar for the United States (panel a)) and the rest of the OECD (panel b)). The percent in pain in the whole populations is constant between ages 56 and 65. So too is the percent of workers in pain. The big changes are the increase in the percentage of people not in work and the falling pain rates among those out-of-work. The percentage in employment declines very quickly between age 56 and 65 in both the United States (from 68% to 39%) and the rest of the OECD (from 72% to 29%). At the same time as the percent non-employed is rising, the pain rate among the non-employed is falling quickly (from 51% at age 56 to 35% at age 65 in the USA, and from 39% to 31% in the rest of the OECD). These patterns can only be explained by the employed shifting into non-employment, taking their relatively low pain incidence with them. The fact that pain rates are constant over this age range among the employed suggests that those leaving for non-employment were not exceptional in terms of their pain rates. The higher pain rates in the United States when people are aged 56–65 can not be related to employment rates–since these are higher in the United States from around age 60 –nor can it be due to pain rates among workers (since these are almost identical). Instead, the difference is due solely to higher pain rates among the non-employed–these are higher than the pain rates among the non-employed in the rest of the OECD throughout the life-course.

It is notable that similar trends are apparent in both the United States and the rest of the OECD when, instead of examining pain rates, we examine the percent of citizens with health problems. The incidence of health problems is roughly constant between ages 56 and 65 in the United States, and rises only very marginally among workers in the rest of the OECD (Table 3). What is striking is the declining rate of health problems experienced by non-workers, a trend which occurs not because existing non-workers are suddenly getting healthier, but because they are being joined by healthier employees switching to non-employment.

**Table 3:** Percent with health problems in the US and OECD’.

	USA		OECD	
	Non-workers	Workers	Non-workers	Workers
56	55	16	47	21
57	55	16	47	20
58	55	16	44	20
59	53	17	46	22
60	49	16	40	22
61	49	17	41	22
62	45	17	41	23
63	44	18	40	22
64	42	17	39	24
65	38	17	38	24

Finally, we switch to a regression context to look at the independent association between suffering pain and various covariates in the United States (Table 4) and the rest of the OECD (Table 5). In both cases we run separate regressions for workers and non-workers.

**Table 4:** OLS pain in the USA, 2009–2017, age 18–65.

	Workers	Non-workers
Age	.0051 (23.92)	.0381 (102.47)
Age^2^*100	-.0035 (14.02)	-.0380 (134.45)
Male	-.0086 (10.89)	.0121 (7.89)
High school graduate	-.0562 (24.98)	-.0902 (31.55)
Technical/Vocational	-.0416 (15.96)	-.0554 (14.34)
Some college	-.0696 (31.80)	-.0936 (33.13)
College graduate	-.1212 (55.69)	-.2137 (70.63)
Postgraduate	-.1363 (61.33)	-.2547 (77.24)
Year	.0037 (21.92)	.0033 (9.87)
FT self-employed	.0284 (19.89)	
PT	.0232 (16.55)	
PT wants FT	.0928 (64.61)	
OLF		.0920 (45.16)
_cons	.1313	-.4663
N	972,834	381,461
Adjusted R^2^	.0206	.0790
Age maximum	73	50

All equations include 50 state dummies. Excluded: FT employee in column 1 and unemployed in column 2; High school dropout.

**Table 5:** OLS pain in the OECD, 2009–2020 age 15–65.

	Workers	Non-workers
Age	.0016 (18.39)	.0212 (26.98)
Age^2^*100		-.0209 (22.19)
Male	-.0153 (7.03)	-.0238 (6.17)
Tertiary	-.0289 (6.65)	-.0539 (8.72)
College graduate	-.0749 (14.39)	-.1289 (18.13)
Year	.0000 (0.29)	.0008 (1.27)
FT self-employed	.0171 (4.80)	
PT	.0282 (8.93)	
PT wants FT	.0539 (13.88)	
OLF		.0539 (10.18)
_cons	-.0346	-1.6722
N	135,980	56,218
Adjusted R^2^	.0152	.0460
Age maximum	n/a	51

All equations include 19 country dummies. Excluded = FT employee in column 1 and unemployed in column 2; High school dropout.

Beginning with the United States in Table 4 it is apparent that pain incidence is an inverted U-shape in age among workers and non-workers. The hump maximizes at age fifty in the case of non-workers. For workers it maximizes outside the range of our data at age 73, so for all intents and purposes the relation is linear for the working population. Pain incidence also falls with education among workers and non-workers, and it has been trending upwards over time for both. Among workers, those in full-time employment suffer the least pain, whilst the underemployed–those who are working part-time but would prefer more hours–suffer most. Among non-workers, those who are out of the labor force suffer considerably more pain than those who are attached to the labor market and are seeking work.

In the rest of the OECD, pain rises linearly with age among workers but is hump-shaped among non-workers (Table 5) and maximizes at age fifty-one. The age squared term for workers is insignificant and omitted. Pain is considerably lower for college graduates than the less-well-educated, both among workers and non-workers, and there has been no trend in pain over time. Similar patterns are apparent across worker-types, with the underemployed reporting greater pain, and among non-workers where those out-of-the-labor force experience greatest pain.

## Discussion

The major finding in Case et al. [1] is that pain is hump shaped in age in the United States for the less educated but rises with age for those with a BA or higher. They show this hump shape is not apparent in the rest of the OECD. Pain rises with age for both the low and high educated. However, we find, by comparing the same pain data by age, but for workers and non-workers, remarkable similarities in the experience of pain in the United States and other OECD countries. Whilst the incidence of pain is high in the United States compared to elsewhere among those without work (Fig 4), the hump-shaped pain profile by age is apparent in both the United States and the rest of the OECD among those without work. Pain is lower and rises gradually with age among workers in both the United States and the rest of the OECD. These patterns are apparent *within* educational groups.

We conclude that what matters in explaining pain over the life-course is whether one is working or not and that this is apparent across countries. In this sense our results reflect Krueger’s [2] study which emphasized the importance of work in understanding patterns in pain across the life-course.

Whereas Case et al [1] pointed to growing pain across cohorts among less educated Americans as a reason for the ‘mystery’ of American pain we have shown the age profile of pain is similar in the United States to elsewhere when we adjust for differences in the age-employment rate profile between the United States and elsewhere. In the United States and elsewhere pain rises with age among workers but is hump shaped among non-workers, maximizing around age 50 and declining thereafter. It is the lower employment rates in the United States, coupled with the much greater pain experienced among the non-employed, that explain much higher pain rates in the United States through age 60. When we change employment rates in the United States they resemble the age profile of pain elsewhere in the OECD.

We are then left with the puzzle: why should pain be hump-shaped among the non-employed after they hit their early 50s in both the United States and the rest of the OECD? Some of the explanation may lie in early death rates of the non-employed which are linked to their high pain incidence. But most of it is likely due to the movement of workers into non-employment as they age. These workers tend to experience much lower pain than non-workers. As workers switch to non-work the increasing size of the ex-worker group pulls down the pain average among non-workers. The non-worker fall in pain, from age 56 to 65 is thus something of an illusion: it is simply likely to reflect compositional change.

Unfortunately, the United States has experienced a substantial decline of over six percentage points in the employment rate in the last two decades, well in excess of other OECD countries (S1 Table). The US employment rate fell from 74% in 2000 to 67% in 2020. In 2005, for example, the United States had the ninth highest employment rate out of the 22 OECD countries in our data. By 2020 it had fallen to 17^th^ position, ahead of France, Belgium, Spain, Italy and Greece. Fixing the American problem of pain requires fixing the labor market for the left-behinds [17].

The employment rate (employment /population or EPOP) rose steadily in the United States from a low of 55.0% in 1961, with occasional peaks and troughs, to a peak of 64.7% in April 2000. The United States entered the Great Recession according to the NBER Business Dating Group in December 2007, when the EPOP was 62.7%. EPOP then fell to a low of 58.2% in 2011. Since then, EPOP has risen steadily through the start of the pandemic in January 2020 to 61.1%—the same as it was at the start of 1987—which is 2.6 percentage points below the level at the start of the Great Recession. Even though EPOP rates fell faster in the United States relative to other countries from 2000, actual hours of work fell less–by 2.9% between 2000 and 2019 –versus, for example, by 5.4% in Germany; 6.5% in Canada. Hours of work in the United States in 2019 remain well above that of 19 of the twenty OECD countries.

Since the onset of Covid-19 in 2020 employment rates have collapsed. In January 2021 the US employment rate was 57.5%, up from 51.3% in April 2020. These rates are comparable to the monthly average of 57.0% for the first quarter century after the Second World War (January 1948-December 1982). The employment rates published by the BLS since April 2020 are upper bound estimates of the actual rates. This has arisen because of misclassification error identified by the BLS relating to employed persons absent from work due to temporary, pandemic-related business closures or cutbacks. Some of the workers affected by the pandemic who should have been classified as unemployed on temporary layoff were instead misclassified as employed but not at work. In April 2020 this error raised the official rate from 14.7% to 19.7% and continued to do so, but by less, in subsequent months. In January 2021 this error lowered the unemployment rate by 0.6 percentage points and many other labor market variables are also impacted including the employment rate which is biased upwards. See https://www.bls.gov/covid19/employment-situation-covid19-faq-january-2021.htm The longer the US employment rate remains low the bigger the potential for pain to rise in future since, as we have shown, joblessness is strongly correlated with pain.

The big fall in employment rates observed in the United States since 2000, relative to competitor countries, was not caused by a big rise in pain. When the economy suffers an economic shock, it is liable to result in an increase in pain–the causal arrow going from work to pain, rather than vice versa, in much the same way as we see in micro-studies which find an increase in unhappiness after job loss [18]. Of course, pain can lead to unemployment, but this doesn’t seem the dominant direction of causation. It seems a negative macroeconomic shock has hit the US which resulted in declining proportion of the adult population in jobs and hence more pain rather than the reverse. This aggregate employment shock has hit the US worse than elsewhere which seems to account for the disproportionate rise in pain. Lack of jobs increased pain mechanically. What also sets America apart are its high levels of income and wealth inequality, lack of health insurance as well as high levels of obesity, which makes it hard for Americans at the low end especially to ride through the shock.

There is little consensus among economists regarding the explanation(s) for the exceptional decline in employment rates in the United States compared to other countries since 2000. A number of possibilities have been floated although many are unconvincing as they also apply to other advanced countries. Aaronson et al (2014) [19] for example, argued that most of the fall is attributable to demographic change. However, these changes are not sizeable enough, nor exceptional enough by international comparative standards, to account for the size of the decline. Canada, Japan, the UK, France and Germany also have aging populations. Technology is common.

A second possibility is that the decline is partly a reversal in the phenomenal employment growth experienced in the previous decade by the US, which arose for unsustainable reasons such as the dotcom ‘bubble’. This ‘regression to the mean’ type argument may, again, be somewhat relevant, but was not so exceptional in the United States when compared to other countries. In the most recent review of the issue Abraham and Kearney (2018) [20] list a range of potential reasons, including the import penetration shock from China. This argument seems appealing yet, even here, the evidence is not so clear. Increased trade with China has been employment enhancing in net terms, despite substantial job loss in the most exposed sectors like manufacturing [21]. One possible explanation for poor US job growth is the relative success it has had in deploying new technologies in the workplace [22] and greater incidence of good management in the United States compared to elsewhere [23].

Most importantly from our perspective, no analysts point to pain levels or trends in pain as a reason for changes in employment rates. The relationship runs dominantly from lack of jobs to pain rather than the reverse. Instead, what is most apparent is that the US economy has become increasingly incapable of creating jobs after recessionary shocks that would be sufficient to return to previous employment rates. This appears as a ratcheting down effect with each recession such that output growth comes without jobs growth. This pattern of jobless growth may be another aspect of a decline in labor’s share–not in wage rates, but in the total quantity of labor used in the economy.

## Materials and methods

We have prepared code, micro data and excel files that can be used to replicate our results. The underlying Gallup data are proprietary and require a subscription either directly or through a university library.

## Supporting information

S1 File Plos one supplementary material. (DOCX)

S1 Table Employment rate, % of working age population, 2000–2020. (DOCX)

S1 Fig Employment rates. (DOCX)
