# Supplementary material for: Further decoding the mystery of American pain: The importance of work
Source: PLoS One. 2022 Jan 13;17(1):e0261891. doi: 10.1371/journal.pone.0261891 (PMC8758074; doi:10.1371/journal.pone.0261891)
Supplement: S1 File — (DOCX) [file pone.0261891.s001.docx]

**Blanchflower and Bryson (2021) “Further decoding the mystery of American pain: the importance of work” – Supplementary Material**

Data on pain prevalence for those aged 65 and under are available in two Gallup surveys, namely the Gallup Daily Tracker Survey (2008-2017) for the United States – henceforth GUSDT – containing 1,354,445 unweighted observations, and the Gallup World Poll (2005-2018) – henceforth GWP - for a further twenty OECD countries containing 194,576 unweighted observations. These countries are: Australia, Austria, Belgium, Canada, Denmark, Finland, France, Germany, Iceland, Ireland, Italy, Japan, Luxembourg, the Netherlands, New Zealand, Norway, Spain, Sweden, Switzerland, and the United Kingdom.

In the various figures following Case et al (2020) [1] we average across the years 2009-2020 for the OECD and 2009-2017 for the USA. There is a longer time run for the OECD20 countries, but we exclude these additional years from 2005-2008 as there is no comparable labor market variable. But we include them in Table 1.

Both have the same short-term retrospective question about the presence and duration of pain: “Did you experience the following feelings during a lot of yesterday? How about physical pain? Yes/No”. They are the two Gallup studies used by Case et al. [1]. However, unlike Case et al. [1] we retain all survey respondents, regardless of race, for those of working age. We confine our analyses to those aged 65 and under given our focus on the role of work.

When focusing on the role of work in the US we also confine analyses to the years 2009-2017 where the work question is consistent. That means we drop 2008 entirely and only retain 12,026/246,728 observations for those age <66 in 2009 that responded to the five category employment variable (employment 2010).

In the OECD for those age 15-65 we have pain data back to 2005 (n=240,287). But we only have consistent employment data back to 2009 for those age<66. Hence, we drop 45,711 observations for 2005-2008.

Throughout we pool all years of data available for the United States and other OECD countries separately.

Supporting information, Fig. 1. pain incidence in GUSDT, 2009-2017 for those aged 18-65 weighted by variable *comb_weight* (n=1,354,445). For GWP 2009-2020 (n=194,576).

Supporting information, Fig. 2: pain incidence in GUSDT, 2008-2017 pooled, by education for those aged 18-65 weighted by variable *comb_weight*. Line graph for percentage reporting pain by age and education for each year of age cell. The imposition of the weights has little effect – mean % reporting in pain unweighted is .235 and weighted .238 (n=1,354,296). Pain rate averages are: Less than high school=38.4%; HS graduate=26.8%; Technical/vocational=28.3%; some college=24.0%; College=16.6% and postgraduate=15.1%.

Supporting information, Fig. 3: pain incidence in GWP, 2009-2020 pooled, by education status, for those aged 18-65. (n=169,604). Pain probabilities are completed secondary or less=28.9%; Secondary – 3-year tertiary=23.4% and college=17.8%.

Supporting information, Fig. 4: pain incidence in GUSTD 2009-2017 and GWP, 2009-2020 pooled, by work status, for those aged 18-64. Pain among workers, with weights imposed for US, workers=18.5% (n=972,929) and 36.6% for non-workers (n=381,516). For OECD averages=19.2% for workers (n=137,651) and 28.6% for non-workers=28.6% (n=56,925).

Supporting information, Fig. 5: pain incidence in GUSTD 2009-2017 and GWP, 2009-2020, for workers aged 18-65. Pain among workers, with weights imposed for US, non-college=21.2% (n=486962) and 13.8% for college (n=477,063). For OECD averages=16.2% for college (n=52,562) and 21.1% for non-college (n=82,658).

Supporting information, Fig. 6: pain incidence in GUSTD 2009-2017 and GWP, 2009-2020, for non-workers aged 18-65. Pain among nonworkers, with weights imposed for US, non-college=39.0% (n=261,789) and 26.3% for college (n=114,237). For OECD averages=25.1% for college (n=12,394) and 29.5% for non-college (n=43,175).

Supporting information, Fig. 7: shift-share analysis of pain in the United States and elsewhere in the OECD. Shows the incidence of pain in the United States and elsewhere in the OECD by age, plus the pain profile by age of Americans when weighting that pain by employment rates for each year of age in the OECD.

Supporting information, Appendix Fig. S1: employment rates by age in GUSDT (2009-2017) and GWS (2005-2018), for those aged 18-65 years pooled.
