# Supplementary material for: Further decoding the mystery of American pain: The importance of work
Source: PLoS One. 2022 Jan 13;17(1):e0261891. doi: 10.1371/journal.pone.0261891 (PMC8758074; doi:10.1371/journal.pone.0261891)
Supplement: S1 Table — (DOCX) [file pone.0261891.s002.docx]

Appendix Table S1. Employment rate, % of working age population, 2000 – 2020

Location 2000 2005 2008 2013 2018 ▾2020

Australia 69.1 71.5 73.2 72.0 73.8 72.7

Austria 68.5 67.4 70.8 71.4 73.0 72.4

Belgium 60.5 61.1 62.4 61.8 64.5 64.7

Canada 70.9 72.4 73.4 72.3 73.5 70.0

Denmark 76.3 75.9 76.3 70.8 74.1 74.4

Finland 67.2 68.4 71.1 68.9 72.1 72.1

France 63.8 64.9 64.1 65.3 65.3

Germany 65.5 70.1 73.5 75.9 76.2

Greece 56.5 59.6 61.4 48.8 54.9 56.3

Iceland 83.8 83.6 81.1 85.1 80.3

Ireland 65.2 67.6 69.7 61.7 68.7 67.8

Italy 53.7 57.6 58.7 55.5 58.5 58.1

Japan 69.0 69.4 70.9 71.8 76.9 77.4

Luxembourg 63.6 63.4 65.7 67.1 67.3

Netherlands 73.0 70.6 74.9 73.6 77.2 77.8

New Zealand 70.3 74.2 74.6 72.7 77.5 76.8

Norway 77.5 74.8 78.0 75.4 74.8 74.7

Spain 56.3 63.6 64.5 54.8 62.4 61.0

Sweden 72.3 74.3 74.4 77.4 75.5

Switzerland 77.2 79.5 78.4 80.1 79.9

United Kingdom 72.5 72.9 72.6 71.5 75.6 75.4

United States 74.1 71.5 70.9 67.4 70.7 67.1

Source OECD The working age population refers to people aged 15 to 64

https://data.oecd.org/emp/employment-rate.htm
