# Supplementary material for: Further decoding the mystery of American pain: The importance of work
Source: PLoS One. 2022 Jan 13;17(1):e0261891. doi: 10.1371/journal.pone.0261891 (PMC8758074; doi:10.1371/journal.pone.0261891)
Supplement: Supplementary file 3 [file pone.0261891.s003.docx]

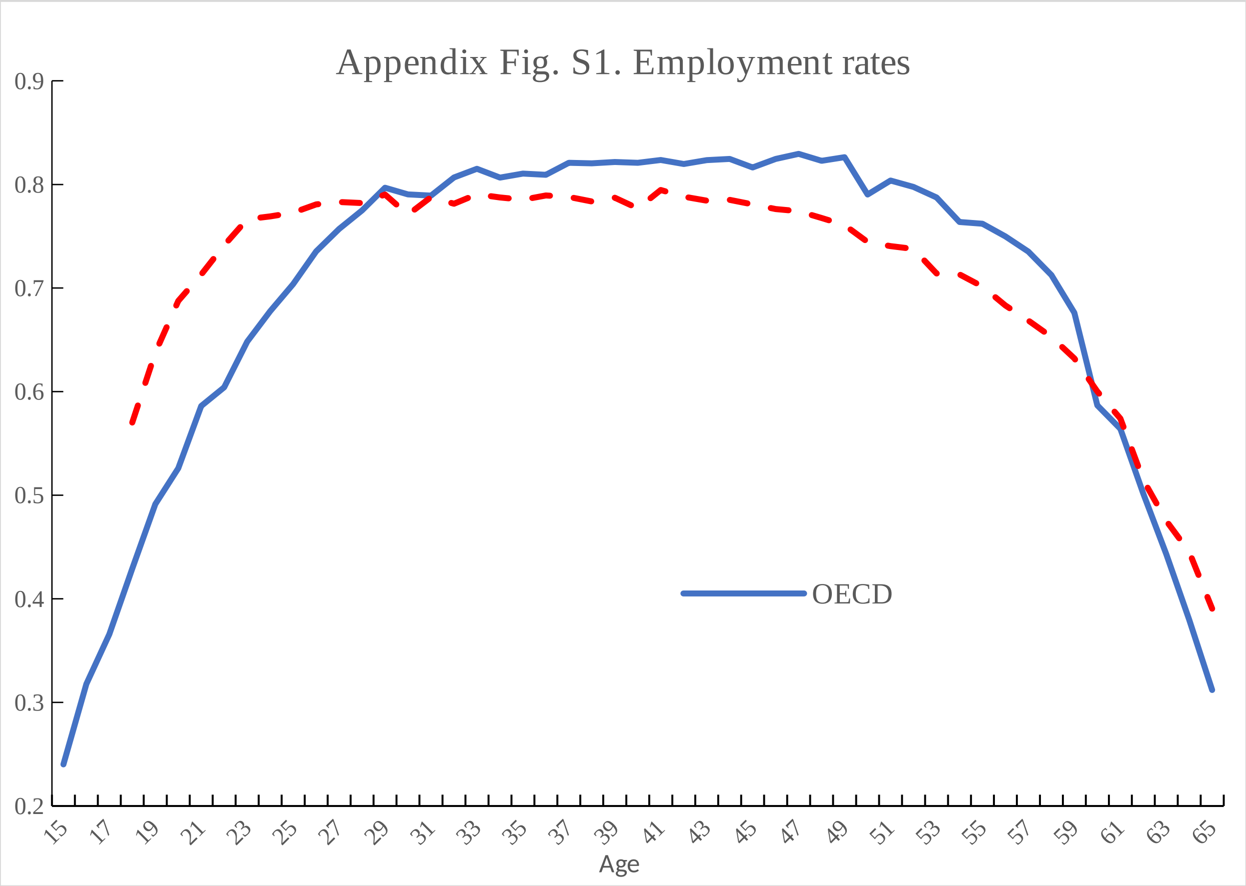
Appendix Fig. S1. Employment rates
0.9
0.8
0.7
0.6
0.5
0.4
0.3
0.2
15
17
19
21
23
25
27
29
31
33
35
37
39
41
43
45
47
49
51
53
55
57
59
61
63
65
Age
OECD
